# Nontoxic Cobalt(III) Schiff Base Complexes with Broad‐Spectrum Antifungal Activity

**DOI:** 10.1002/chem.202003545

**Published:** 2020-11-24

**Authors:** Angelo Frei, A. Paden King, Gabrielle J. Lowe, Amy K. Cain, Francesca L. Short, Hue Dinh, Alysha G. Elliott, Johannes Zuegg, Justin J. Wilson, Mark A. T. Blaskovich

**Affiliations:** ^1^ Centre for Superbug Solutions Institute for Molecular Bioscience The University of Queensland St. Lucia QLD 4072 Australia; ^2^ Department of Chemistry and Chemical Biology Cornell University Ithaca NY 14853 USA; ^3^ Department of Molecular Sciences Macquarie University Sydney NSW 2109 Australia; ^4^ Department of Biological Sciences Macquarie University Sydney NSW 2109 Australia

**Keywords:** antifungal agents, cobalt, metallodrugs, Schiff base complexes

## Abstract

Resistance to currently available antifungal drugs has quietly been on the rise but overshadowed by the alarming spread of antibacterial resistance. There is a striking lack of attention to the threat of drug‐resistant fungal infections, with only a handful of new drugs currently in development. Given that metal complexes have proven to be useful new chemotypes in the fight against diseases such as cancer, malaria, and bacterial infections, it is reasonable to explore their possible utility in treating fungal infections. Herein we report a series of cobalt(III) Schiff base complexes with broad‐spectrum antifungal activity. Some of these complexes show minimum inhibitory concentrations (MIC) in the low micro‐ to nanomolar range against a series of *Candida* and *Cryptococcus* yeasts. Additionally, we demonstrate that these compounds show no cytotoxicity against both bacterial and human cells. Finally, we report the first in vivo toxicity data on these compounds in *Galleria mellonella*, showing that doses as high as 266 mg kg^−1^ are tolerated without adverse effects, paving the way for further in vivo studies of these complexes.

## Graphical abstract

**Metals vs. fungi**: Given that metal complexes have proven to be useful new chemotypes in the fight against diseases such as cancer, malaria, and bacterial infections, it is reasonable to explore their possible utility in treating fungal infections. Herein we report a series of cobalt(III) Schiff base complexes with broad‐spectrum antifungal activity against *Candida* and *Cryptococcus* yeasts, with MICs in the low micro‐ to nanomolar range.

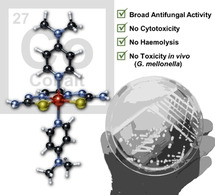

## Introduction

The human body is well equipped to fight off most primary fungal infections as the combination of high body temperature and a sophisticated immune system make it difficult for fungi to colonise the human body. However, an unfortunate side effect of many medical advances such as chemotherapy, transplants requiring immunosuppressive therapy, and broad‐spectrum antimicrobial drug treatments has been the induction of higher susceptibility to fungal infections in patients.^[1]^ The human immunodeficiency virus (HIV) epidemic has facilitated many fungal infections. At its height in the early 2000s, an estimated 600 000 people died of HIV‐related opportunistic cryptococcal meningitis each year.^[2]^ Although this number has since been trending downward, the mortality of fungal infections remains high, with an estimated 220 000 cases of cryptococcal meningitis among people with HIV/AIDS worldwide in 2017, resulting in nearly 181,000 deaths.^[3]^ In the meantime, drug‐resistant fungi are on the rise. Amongst these, *Candida auris* is particularly worrisome as its morbidity around the world is rising and the organism is showing resistance to the current arsenal of antifungal drugs, leading to high mortality rates.^[4]^ Despite these looming threats, the antifungal drug pipeline is even sparser than the notoriously depleted antibacterial portfolio, with fewer than ten antifungal compounds with novel targets in clinical trials as of 2018.^[1]^ To prevent antifungal resistance from becoming a global health crisis, new drugs with novel modes of action are required.

Metal complexes have been studied extensively for anticancer applications, with drugs such as cisplatin still widely used in chemotherapy today.^[5]^ Many metal compounds are currently under clinical investigation for anticancer therapy^[6]^ and their applications are being extended to other diseases such as malaria^[7]^ and neurodegenerative diseases,^[8]^ helping to decrease the perception that metal complexes are inherently toxic. However, antimicrobial applications,^[9]^ particularly use as antifungals, have largely been ignored by the inorganic medicinal chemistry research community to date.^[10]^

**Figure 1:**
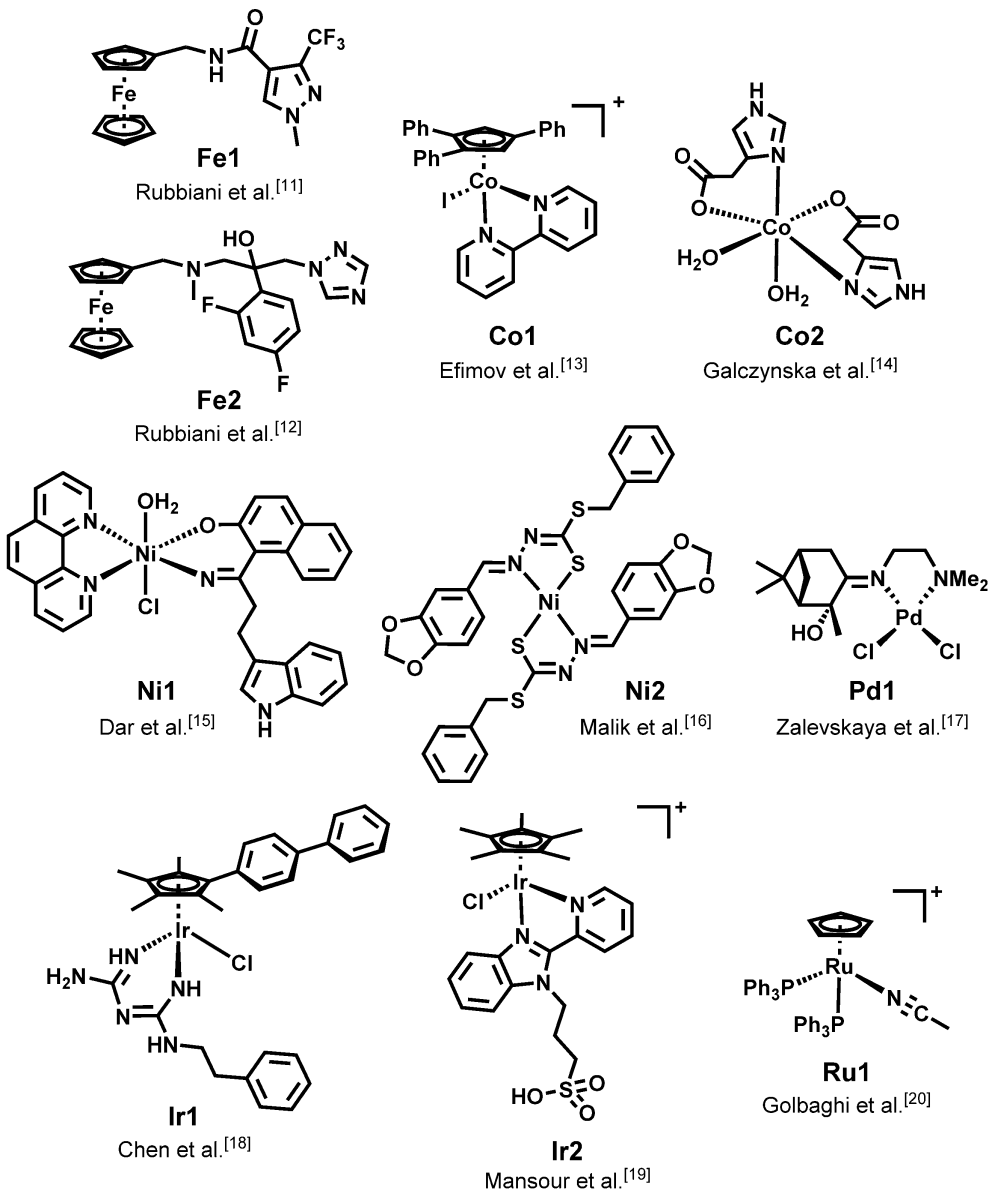
Structures of metal complexes with reported antifungal activities.

In 2016, Rubbiani, Gasser and co‐workers introduced ferrocenyl and ruthenocenyl moieties into the organic scaffold of the broad‐spectrum fungicide sedaxane. One ferrocene (**Fe1**, Figure 1) derivative was found to display modest antifungal activity against *Saccharomyces cerevisiae* (EC_50_=43 μM) while no cytotoxicity against human cell lines was found up to 100 μM.^[11]^ Just before this manuscript was submitted, Rubbiani and Gasser published a preprint article on a new class of organometallic fluconazole derivatives (e.g., **Fe2**, Figure 1). These ferrocene‐bearing compounds were shown to have improved antifungal activity against *C. robusta* relative to fluconazole. The authors demonstrated that the metal‐bearing derivatives could still effectively inhibit lanosterol 14α‐demethylase, the same enzyme that is targeted by fluconazole. The best compound (**Fe2**) showed MIC_50_ values in the nanomolar range against several fungi, including fluconazole‐resistant strains. Finally, a first in vivo mouse study of the compound was described. Treatment of mice bearing a *Candida* infection with 10 mg kg^−1^ of **Fe2** did not decrease fungal burden in the kidney. However, the treatment seemed to significantly improve the inflammatory pathology in the kidney and colon relative to untreated mice.^[12]^

In other work, Efimov et al. described a series of half‐sandwich cobalt(III) and iron(III) complexes and found that only compounds bearing a metal‐iodine bond (e.g., **Co1**) showed significant activity against a panel of six phytopathogenic fungi at 30 μg mL^−1^. However, no activity at lower concentrations or other toxicity data were reported. Nevertheless, these compounds represent a new class of half sandwich complexes and warrant further investigations.^[13]^

Gałczyńska et al. reported a series of copper(II) and cobalt(II) complexes with the ligands imidazole‐4‐acetate (iaa) and 1‐allylimidazole and found that the Co^II^‐iaa compound (**Co2**) showed a significant decrease (≈5 log) in colony forming units (CFU) against *Candida albicans* at 60 μM in vitro, while not displaying any cytotoxicity at the same concentration. Unfortunately no dose‐response or activity at lower concentrations were reported.^[14]^ In 2019, Hashmi and co‐workers described the preparation of Co, Ni, Zn, and Cu complexes bearing a 1,10‐phenanthroline ligand as well as a Schiff‐base‐derived ligand with a pendent indole ring (e.g., **Ni1**). The cobalt and nickel complexes showed high antifungal activity against a series of *C. albicans* strains (including some fluconazole‐resistant isolates), with MICs ranging from 0.25‐8 μg mL^−1^. However, no studies against other fungal pathogens or cytotoxicity against human cell lines were reported.^[15]^ The same group more recently revealed another series of Cu, Co, and Ni complexes with *S*‐benzyldithiocarbazate imine ligands. Again, the cobalt and more so, the nickel compounds (e.g., **Ni2**) displayed good activity against a wide spectrum of *C. albicans* isolates. Notably, the activity against fluconazole‐resistant isolates was significantly decreased relative to the fluconazole‐susceptible strains (MIC_resistant_=8–256 μg mL^−1^, MIC_susceptible_=0.5–64 μg mL^−1^). While no data on cytotoxicity were presented, the authors conducted haemolysis studies with the compounds on horse red blood cells. Cell haemolysis between 8–12 % was detected for the metal complexes at MIC concentrations. A concentration dependency of the haemolysis was found, suggesting the potential for adverse effects of these compounds at higher concentrations.^[16]^

In 2020, Zalevskaya et al. described the antimicrobial properties of terpene‐derived palladium complexes. Many of the compounds showed activity against both methicillin‐resistant *Staphylococcus aureus* (MRSA) as well as *C. albicans and C. neoformans*. Unfortunately, most compounds also possessed some degree of cytotoxicity and/or caused haemolysis. On the other hand, complex **Pd1** showed MICs of 38.5 μM (16 μg mL^−1^) and 4.8 μM (2 μg mL^−1^) against *C. albicans* and *C. neoformans*, respectively while not showing any toxicity or haemolysis up to 77.0 μM (32 μg mL^−1^).^[17]^ The group of Sadler reported on the impressive antimicrobial profile of a series of iridium(III) biguanine half‐sandwich complexes in 2018. Besides the excellent broad‐spectrum antibacterial and antibiofilm activity of some of the compounds, the group also reported antifungal activity (e.g., **Ir1**) that was up to 76 times higher against *Cryptococcus neoformans* when compared with the control drug, fluconazole.^[18]^ A similar class of compounds was studied by Mansour et al. for their antimicrobial properties. While the compounds with antibacterial activity also displayed some degree of cytotoxicity, one compound (**Ir2**) was nontoxic and nonhaemolytic (CC_50_ and HC_10_≥44.8 μM) while displaying an MIC of 11.2 μM against *C. neoformans*.^[19]^ Golbaghi et al. reported two ruthenium half‐sandwich complexes with activity against a range of *Candida* species (MIC=2.4–5.6 μM, **Ru1**). It was found that the positive charge of the complexes was essential for the activity as the neutral complex (with a chloride instead of the acetonitrile ligand) showed no antifungal activity up to 20 μM. Remarkably, the authors could show that the cellular uptake of the ruthenium complexes, as tracked by ICP‐MS, correlated well with both ROS generation and antifungal activity. These findings were supplemented by docking studies suggesting that the metal complex can interact with the CYP51 enzyme more favourably than the commercial antifungal drug fluconazole (the primary target of azole antifungal drugs is fungal lanosterol 14α‐demethylase, which belongs to the CYP51 class of cytochrome P450 enzymes).[^20^, ^21^] These findings suggest that these ruthenium complexes could act via more than one mechanism, ROS generation and CYP51 inhibition, decreasing the potential for fungi to develop resistance. Unfortunately, no data on toxicity against mammalian cells was reported for these complexes, preventing any conclusions about their possible in vivo applications.

In a global collaborative effort, our group recently reported the analysis of the antimicrobial properties of 906 metal complexes within the collection of the Community for Open Antimicrobial Drug Discovery (CO‐ADD). We found that metal complexes had a 10× higher hit‐rate against critical bacterial ESKAPE pathogens and yeasts (fungi), compared with the 300 000 organic compounds in the collection, while displaying similar rates for cytotoxicity and haemolysis. The analysis identified 88 active metal complexes without cytotoxicity or haemolytic activity (at concentrations up to 32 μg mL^−1^), 71 of these displayed activity against at least one of the two yeasts tested, *C. albicans* and *C. neoformans*.^[22]^

Metal complexes are intriguing drug candidates because they provide unique three‐dimensional structural diversity^[23]^ and have access to novel modes of action that are not possible with organic molecules.^[24]^ Altogether, while very sparse, the recent literature clearly points to a remarkable potential for antifungal drug development in the realm of metal complexes.

Herein we report on our continued investigation into a class of Schiff‐base cobalt(III) complexes and their antifungal activity, with structure–activity relationship data against *Candida* and *Cryptococcus* strains, cytotoxicity and haemolytic activities. The studies identified several metal complexes with excellent activity against multidrug‐resistant strains with good therapeutic index and no detectable toxicity in an in vivo moth animal model, identifying metal complexes with potential for further drug development.

## Results and Discussion

**Figure 2:**
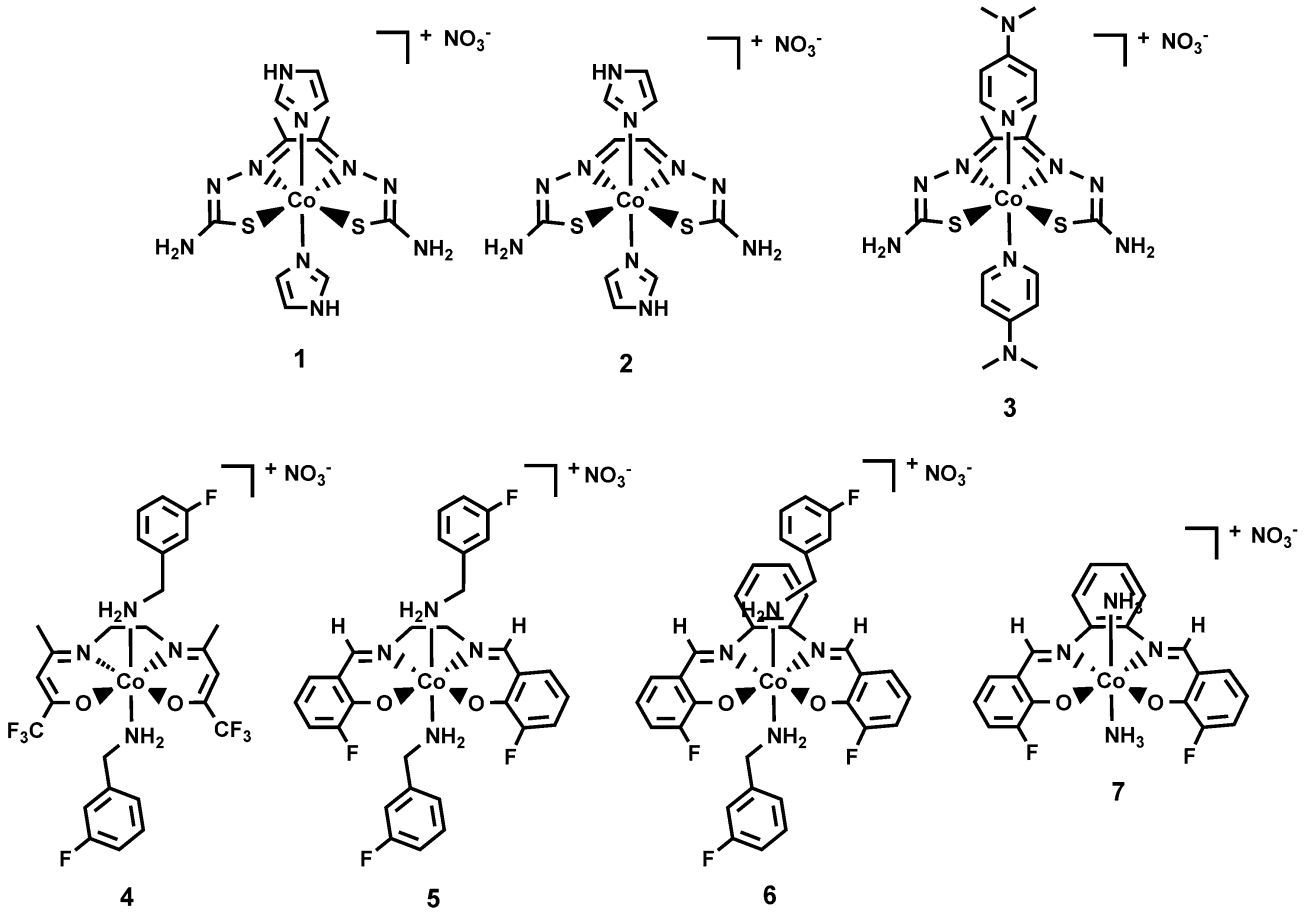
Structures of cobalt complexes **1**–**7**.

The cobalt complexes **1**–**7** (Figure 2) reported in this work were prepared according to a simple general reaction scheme reported previously.^[25]^ Briefly a solution of Co(NO_3_)_2_ is treated with an equimolar solution of the desired equatorial ligand in methanol, and the mixture is heated at reflux for an hour to generate an uncharacterised Co^II^‐intermediate. The axial ligand is then added and the mixture is stirred for at least another two hours at reflux in the presence of air, resulting in the precipitation of the desired Co^III^ product. The compounds were characterised by ^1^H‐NMR spectroscopy, HRMS, IR spectroscopy and elemental analysis. Complexes **3**, **6**, and **7** are previously unreported compounds, and their characterisation data is reported in the supplemental information. We chose to synthesise **6** and **7** as we hypothesised that they would serve as more hydrophobic analogues of **4** and **5**, which might increase their biological activity by facilitating cellular uptake. We synthesised complex **3** as previous studies have shown that very labile complexes are more active in biological systems, as explained in further detail below. In previous work it has been established that these types of Co^III^ complexes undergo ligand‐exchange reactions in aqueous solution where the two axial ligands are sequentially exchanged with neutral donors such as imidazole or water.[^25^, ^26^]

**Figure 3:**
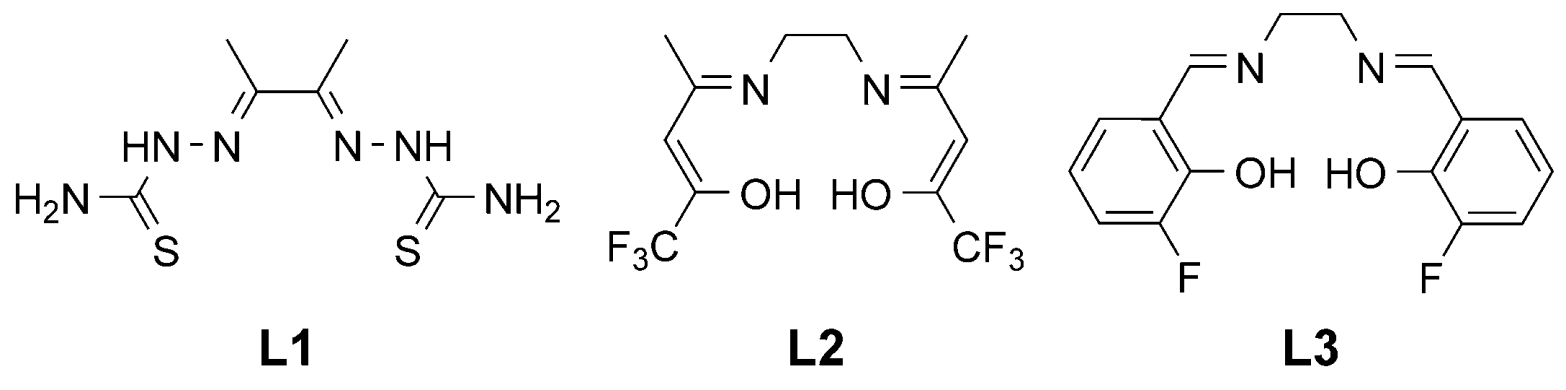
Structures of Schiff‐base ligands **L1**–**L3**.

The stability of this family of compounds toward axial ligand substitution varies widely based on the ligand scaffolds employed. Complexes bearing strongly donating equatorial ligands, such as the bis(thiosemicarbazones), undergo rapid axial ligand substitution, with substantial reactivity observed within minutes at 37 °C.^[25a]^ By contrast, complexes with more weakly donating equatorial ligands, such as **L2** (Figure 3), are inert toward ligand substitution, with half lives of several hours toward substitution by *N*‐methylimidazole at 37 °C.^[25b]^ The properties of the axial ligands are also important, as more strongly donating axial ligands, such as ammonia have longer half‐lives than weak or sterically hindered axial donors, such as benzylamine.

The reactivity of these Co^III^ complexes toward ligand substitution is also related to their redox activity.^[25]^ Our previous experiments have shown that more easily reduced complexes are generally more active toward ligand substitution, probably because stronger electron donors stabilise the Co^III^ oxidation state and generally lead to more inert complexes. Furthermore, when ligand exchange reactions are carried out in the presence of a reducing agent, such as ascorbic acid, the rate of product formation increases remarkably. Kinetic analysis of the reaction of Co^III^‐Schiff base substitution reactions in the presence of ascorbate showed that the first ligand exchange step occurs at the same rate in both the presence and absence of ascorbate, while the second ligand exchange step to form the final product is greatly accelerated. Based on these results, it appears that the mono‐substituted Co^III^ complexes formed after the loss of one axial ligand are easily reduced, leading to a labile Co^II^ intermediate that can react quickly with other ligands. The electrochemistry and ligand exchange reactions of these complexes are also related to their biological activity, as the more easily reduced, labile complexes were found to possess the highest cytotoxicity toward cancer cells. Together, these studies indicate a biological mechanism wherein the original [Co^III^(chelate)(L)_2_]^+^ complex undergoes ligand substitution in biological media, probably by solvent, to yield a [Co^III^(chelate)(L)(H_2_O)]^+^ compound that may then be reduced to form a biologically active, labile [Co^II^(chelate)(H_2_O)_2_] complex.

Because our previous results had shown that complexes bearing weak axial donors are more biologically active, we sought to synthesise a very labile Co^III^‐bis(thiosemicarbazone) complex by using a weakly donating pyridine axial monodentate ligand. We were unable to synthesise a complex using unsubstituted pyridine, probably because it is a such a weak donor ligand that it will not remain bound to the Co^III^ center even briefly, preventing isolation of the complex. However, we were able to isolate a complex (**3**) bearing the more electron‐donating 4‐(dimethylamino)pyridine (DMAP) ligand in the axial positions. As expected, this complex is extremely labile. Upon dissolution in water at room temperature the compound immediately yields a mixture of species, as determined by ^1^H NMR analysis (See Figure S9). Upon incubation overnight at room temperature, the complex becomes totally converted into the bis‐aqua complex, indicating that this compound is the most labile Co^III^ complex we have synthesised to date. In our hands, the bis‐aqua complex could not be isolated.

The electrochemistry of the newly synthesised complexes **3**, **6**, and **7** was investigated so that their properties might be compared with those of the previously reported compounds. Cyclic voltammograms (CVs) for all three complexes are shown in Figures S13–15, and the reduction potentials for all complexes are reported in Table 1. The CVs of these complexes are typical of those reported previously for this class of compounds.[^25^, ^27^] Upon scanning reductively, the Co^III^ complexes first undergo an irreversible, metal‐based reduction to [Co(Schiff base)(L)_2_], which then loses the monodentate axial ligands. Further, reversible reduction yields [Co(Schiff base)]^−^, which can then be oxidised once to [Co(Schiff base)]^+^ and then oxidised again to yield [Co(Schiff base)(Solvent)_2_]^+^. The reduction potentials observed for all three compounds are generally consistent with those reported previously. For compound **3**, the first reduction event (Co^III^/Co^II^) is more positive than that reported previously for compounds **1** and **2**, which is in agreement with the expected lower electron‐donating strength of the DMAP ligand relative to imidazole. For compounds **6** and **7**, the reduction potentials for both redox events are almost identical to those of **4** and **5**, indicating that the fluorosalophen equatorial ligand is almost equivalent in electron donating strength to the unconjugated fluorosalen ligand. For all of the complexes, the first reduction occurs at approximately −0.6 to −0.9 V vs. SCE and the second occurs at around −1.1 to −1.3 V vs. SCE. Thus, only the first reduction event is facile enough to occur under normal biological conditions, and might serve as a potential activation pathway for these compounds (Figure 2).

**Table 1:** Electrochemical properties of the cobalt complexes investigated in this work.

Compound	First reduction *E*_pc_ (V vs. SCE)	Second reduction *E*_1/2_ (V vs. SCE)
**1** ^[a]^	−0.86	−1.19
**2** ^[a]^	−0.72	−1.00
**3** ^[b]^	−0.69	−1.17
**4** ^[c]^	−0.67	−1.31
**5** ^[c]^	−0.58	−1.10
**6** ^[b]^	−0.59	−1.06
**7** ^[b]^	−0.75	−1.08

[a] CV measured in acetonitrile with 0.1 M TBAP.^[25a]^ [b] Measured in DMF with 0.1 M TBAP (this work). [c] Measured in DMF with 0.1 M TBAP.^[25b]^

As previously mentioned, complexes **1**, **2** and **4** were studied in earlier biological assays. Of note, complex **1** displayed no anticancer activity up to 500 μM while **2** showed high activity against cervical cancer HeLa (IC_50_=7.4±2.4 μM) and A549 lung cancer cell lines (IC_50_=12±1.4 μM) under normoxic conditions, but no activity (up to 250 μM) was observed against MRC‐5 normal lung fibroblasts, a noncancerous cell line.^[25a]^ Complex **4** possessed an IC_50_ of 60±17 μM against A549 cells and a high cellular uptake was observed by ICP‐MS.^[25b]^ In our CO‐ADD screening, using broth microdilution (BMD) MIC assays (Table S2), complexes **1**–**3** were effective at killing drug‐susceptible yeast reference strains of *C. albicans* and *C. neoformans* while displaying no antibacterial activity. Conversely, compounds **4**–**6** displayed moderate antibacterial activity against methicillin‐resistant *S. aureus* (MRSA) (MIC(**4**)=4 μg mL^−1^ (5.9 μM), MIC(**5**)=16 μg mL^−1^ (24.7 μM), MIC(**6**)=8 μg mL^−1^ (11.5 μM)), while displaying no antifungal activity against *C. albicans* and *C. neoformans*. Complex **7** showed neither antibacterial nor antifungal activity (Table S2).

Encouraged by these initial results we decided to more closely investigate the antifungal properties of this compound series. We determined the MICs of **1**–**7** as well as ligands **L1–L3** (Figure 3) and Co(NO_3_)_2_ against an extended panel of eight *Candida* and *Cryptococcus* strains. The broader fungal panel encompassed strains with varying resistance profiles, including multidrug‐resistant (MDR) clinical isolates, to better understand the extent of their antifungal activity.

The antifungal MICs are summarised in Table 2, with each entry given as a range of MICs (μM) determined from four datapoints over two biological replicates (*n*=8, equivalent table with values in μg mL^−1^ in Supplementary Information). Three reference antifungal drug compounds were measured alongside the cobalt complexes: the azoles fluconazole (**FCZ**) and ketoconazole (**KCZ**), and the echinocandin micafungin (**MFG**). All compounds showed some activity against at least one yeast strain, with complexes **1**–**3** showing the broadest fungicidal activity spectrum. Complexes **1** and **3** displayed potent MIC values between 0.39–1.56 μM against *C. albicans*, *Candida auris, Candida tropicalis, Cryptococcus deuterogattii,* and *C. neoformans*. Both compounds showed minimal to no activity against *Candida glabrata* (MIC(**1**)=100 μM, MIC(**3**)=50 μM). Of the panel of yeast strains tested, *C. glabrata* is the most intrinsically resistant strain to antifungals, with the exception of echinocandins, and is genetically dissimilar to other *Candida* spp., thus the disparity of activity is somewhat expected.^[28]^ Complex **2** showed a similar activity profile, with a slightly higher MIC range between 0.78–6.25 μM yet more pronounced activity than **1** and **3** against *C. glabrata* (MIC=12.5–25 μM). The near identical MICs for **1** and **3** suggest that the nature of the Schiff‐base ligand is more decisive for the activity profile than the axial ligand. This is consistent with previous findings that the axial ligand is exchanged rather rapidly in solution, a step that might even be necessary for the compound to either enter the target cells or reveal the active molecular component. From these results it seems that the bis‐aqua Co^II^ complex may be the active antifungal species, with the axial ligand acting more as a function to stabilise the precursor complex before dissolution and not contributing or detracting from the antifungal activity. The small but significant difference in activity between **1** and **2** indicates that the structure of the Schiff‐base ligand influences the activity profile, and that further structure–activity relationship investigations should be focused on exploring different variations of this ligand class. However, it is definitely not the ligand alone that accounts for the observed activity, as **L1–L3** (Figure 3) had no detectable antifungal properties (up to 100 μM, Table 2) when tested on their own. Similarly, Co(NO_3_)_2_ when tested alone did not achieve any detectable effect on the growth of the yeast up to 100 μM. This illustrates once more that metal complexes are not merely the sum of their parts but act as new entities with properties that are entirely different from their precursors. It is notable that compounds **1**–**3** have a broader antifungal activity spectrum than the comparator antifungal compounds, underlining their potential as effective antifungals.

**Table 2:** Antifungal activity displayed as minimum inhibitory concentrations (MIC, μM) of all compounds in this study against a panel of fungal strains.

Compd	*C. albicans*	*C. auris*	*C. auris*	*C. glabrata*	*C. tropicalis*	*C. deuterogattii*	*C. deuterogattii*	*C. neoformans*	Tox[a]	Haem[b]
	ATCC 90028	CBS 10913	CBS 12373	ATCC 90030	ATCC 750	CBS 7750	ATCC 32609	ATCC 208821	CC_50_	HC_10_
**1**	0.78–1.56	0.39–0.78	0.39–0.78	100	0.39	1.56	0.78–1.56	0.39–0.78	>100	>100
**2**	1.56–6.25	1.56–3.12	3.12	12.5‐25	0.78–1.56	6.25	3.12	3.12–6.25	>100	>100
**3**	1.56	0.39–0.78	0.78	50	0.39–0.78	1.56	1.56–3.12	0.78–3.125	>100	73.1±20.4
**4**	>100	6.25	100	>100	12.5‐25	50	25	25‐50	>100	>100
**5**	100	12.5	12.5	100	25‐50	12.5‐25	12.5‐25	50	>100	>100
**6**	100	0.098–0.78	25	>100	>100	>100	100	100	>100	>100
**7**	50–100	0.78–1.56	50	100	100	100	100	100	>100	>100
**L1**	>100	>100	>100	>100	>100	>100	>100	>100	>100	>100
**L2**	>100	>100	>100	>100	>100	>100	>100	>100	>100	>100
**L3**	>100	>100	100	>100	>100	>100	100	100	>100	>100
Co^[c]^	>100	>100	100	>100	>100	>100	>100	>100	>100	>100
FCZ^[d]^	6.53	52.2	>209	>209	>209	26.1	26.1–52.2	52.2	n.d.	n.d.
KCZ^[e]^	≤0.94	≤0.94	3.76	60.2	30.1	≤0.94–1.88	1.88	1.88–3.76	n.d.	n.d.
MFG^[f]^	≤0.39	≤0.39	≤0.39	≤0.39	≤0.39	>49.5	>49.5	>49.5	n.d.	n.d.

[a] Cytotoxicity against HEK293 cells. [b] Concentration at which 10 % haemolysis is reached; *n*=8 (two biological replicates in quadruplicate); n.d.: not determined. [c] Co(NO_3_)_2_. [d] Fluconazole. [e] Ketoconazole. [f] Micafungin.

For complexes **6** and **7**, a highly selective activity against the *C. auris* strain CBS 10 913 was found, with MICs as low as 98 nM and 780 nM respectively. No significant inhibitory growth effect was detected against the other strains except for moderate activity against *C. auris* strain CBS 12373, which is less sensitive to antifungal azoles than *C. auris* CBS 10913. The highly specific activity of these complexes is notable and warrants further investigations.

To verify if the potent activity of this compound class is selective for yeasts over mammalian cells, we investigated the toxicity of all compounds by their inhibition of growth of human embryonic kidney epithelial cells (HEK293) and their haemolytic properties against human red blood cells. None of the tested complexes and ligands showed cytotoxicity against HEK293 cells or haemolysis up to 100 μM, with the exception of **3** which showed an HC_10_ (concentration causing 10 % haemolysis) of 73.1 μM ±20.4 μM (Table 2). However, 50 % haemolysis was not reached with any of the compounds at the highest concentrations tested. These results strongly suggest that compounds **1** and **2** are highly selective against yeasts, with no activity against bacterial or human cells.

To further validate the specificity and safety of these compounds, and to substantiate our hypothesis that metal‐containing compounds are not by default harmful to living systems, we advanced compounds **1**–**3** to an in vivo assessment of toxicity. Rodents such as rats and mice are often used for toxicity testing, but these animal models are costly and should be used sparingly for ethical reasons. We therefore opted to conduct first experiments in the greater wax moth *Galleria mellonella*, an insect whose larvae have been used for antimicrobial toxicity studies previously.^[29]^ Toxicity data from this low cost animal model has been shown to correlate well with data from rodent models.^[29b]^ Compounds **1**–**3** were dissolved in DMSO and diluted to final concentrations of 1 μM, 100 μM and 10 mM. For each concentration five larvae (of 200–250 mg in size) were injected with 10 μL and monitored for 96 hours for survival and health using the *G. mellonella* Health Index Scoring System.^[30]^ These experiments were repeated three separate times. All larvae were alive and fully active, displaying full cocoon formation, after the four days, even at the highest concentration. This indicates that the cobalt complexes are nontoxic in vivo up to 10 mM, which equates to an average one‐time dosage of 266 mg kg^−1^. Of note, at the highest concentration, the normally cream/translucent moths exhibited a pinkish colour after compound injection but showed no other signs of detrimental health effects. These findings suggest that compounds **1**–**3** are not harmful to living animals, even at exceedingly high doses, paving the way for in vivo efficacy studies for fungal infections and pharmacokinetics and pharmacodynamics studies.

## Conclusion

The rise of antifungal resistance has been somewhat overshadowed with our attention directed at the current antibacterial antibiotic crisis. However, the decrepit antifungal drug pipeline should be cause for concern and new antifungal drugs with novel modes of action are urgently needed. Metal complexes have displayed increasing potential in many areas of medicine but have only sparsely been investigated against fungal pathogens.

In this work we have shown that cobalt complexes display excellent activity against a panel of relevant fungal strains. Compounds **1**–**3** showed an activity spectrum that is superior to commonly employed antifungal drugs. Furthermore, these cobalt complexes showed no toxicity against human cells at concentrations up to 100 μM, and almost all showed no signs of haemolysis of red blood cells (**3** induced mild haemolysis of red blood cells at higher concentrations, HC_10_=73.1±20.4 μM). The most active compounds were highly selective for yeasts, displaying no activity against any of the bacteria tested. Lastly, we studied the toxicity of compounds **1**–**3** against *G. mellonella*, an established in vivo moth model. The compounds proved safe, with no toxicity observed even at very high doses. While the mechanism of action of these compounds is unknown at this stage, a few deductions can be made from the available data. The ligand exchange and redox behaviour of similar complexes suggest that in solution, one of the axial ligands is substituted rapidly, followed by reduction of the metal center. Only then is the second axial ligand substituted as well. The fact that the free ligands display no activity at all indicates that an intact cobalt‐ligand moiety is required for at least for the uptake into the fungal cells. At this stage it is not clear if the ligand disassociates in the cytoplasm of the yeast cell. Altogether these compounds display excellent activity against a broad range of pathogenic yeast strains while showing no in vitro cytotoxicity and, importantly, no in vivo toxicity, making them a promising starting point for further preclinical investigations.

## Experimental Section

**Materials and methods**: Synthesis of all cobalt complexes was carried out using modifications of previously reported procedures. 3‐fluorosalicylaldehye, 3‐fluorobenzylamine (3F‐BnNH_2_), *o*‐phenylenediamine, thiosemicarbazide, diacetyl, and Co(NO)_3_⋅(6H_2_O) were of reagent grade and were obtained from commercial vendors. Solvents were of ACS grade or higher. The synthesis of the ligands 3‐fluorosalicylaldehyde *o*‐phenylendiamine (3F‐salophen)^[31]^ and diacetyl thiosemicarbazone (ATS)^[32]^ were performed according to literature procedures. The cobalt complexes **1**, **2**, **4**, and **5** were previously synthesised and existing materials were used for these studies.^[25]^ The synthetic details for previously unreported cobalt complexes are outlined below.

**Physical measurements**: NMR spectra were acquired on a 500 MHz Bruker AV 3HD‐spectrometer equipped with a broadband Prodigy cryoprobe or a 400 MHz Varian spectrometer equipped with an auto‐switchable probe. High‐resolution mass spectrometry (HRMS) was performed with a Bruker MicroTOF mass spectrometer using (+)‐ESI calibrated to sodium formate. Samples for IR spectroscopy were prepared as KBr pellets and were analyzed on a Nicolet Avatar 370 DTGS (ThermoFisher Scientific, Waltham, MA).

**[Co(3F‐salophen)(3F‐BnNH_2_)_2_]NO_3_ (6)**: 3F‐salophen (160 mg, 0.65 mmol) was dissolved in isopropanol (15 mL) and Co(NO_3_)_2_⋅6 H_2_O (190 mg, 0.65 mmol) was added, resulting in a green suspension that slowly turned reddish orange. The mixture was heated at reflux for 1 h, after which 3F‐BnNH_2_ (0.5 mL, 4.4 mmol) was added. The mixture was stirred at room temperature overnight, filtered, and the precipitate was washed with isopropanol (20 mL) and diethyl ether (20 mL) to yield 210 mg (45 %) of a dark red powder. Anal. calcd for C_34_H_28_CoF_4_N_4_O_2_⋅NO_3_ (%): C, 56.6; H, 3.91; N, 9.71. Found: C, 56.37; H, 4.09; N, 9.53. ^1^H NMR (500 MHz, [D_6_]DMSO) *δ*=8.86 (s, 2 H), 8.44–8.28 (m, 2 H), 7.48 (s, 4 H), 7.32 (dd, *J*=12, 11 Hz, 2 H), 7.15 (q, *J*=7.6 Hz, 2 H), 6.94 (td, *J*=8.7, 2.3 Hz, 2 H), 6.83 (d, *J*=9.6 Hz, 2 H), 6.73 (d, *J*=7.9 Hz, 2 H), 6.67–6.60 (m, 2 H), 4.15 (t, *J*=6.3 Hz, 4 H), 3.11 (t, *J*=6.3 Hz, 4 H). ^19^F NMR (470 MHz, [D_6_]DMSO) *δ*=−113.42—−113.54 (m, 2F), −134.22 (d, *J*=11 Hz, 2F). IR (KBr, cm^−1^): 3070 br w, 1613 s, 1547 w, 1440 m, 1317 m, 1187 m, 1078 w, 904 w, 878 m, 723 s, 530 m. HRMS‐ESI (positive ion mode, CH_3_CN): *m*/*z* calcd for [C_34_H_28_CoF_4_N_4_O]^+^: 659.1475; found: 659.1490.

**[Co(3F‐salophen)(NH_3_)_2_]NO_3_ (7)**: 3F‐salophen (160 mg, 0.65 mmol) was dissolved in methanol (8 mL) and Co(NO_3_)_2_⋅6 H_2_O (190 mg, 0.65 mmol) was added, resulting in a green suspension that slowly turned black. The mixture was heated at reflux for 1 h, after which 30 % aqueous NH_4_OH (1 mL) was added. The reflux was continued for 16 h, and the resulting orange suspension was allowed to cool to room temperature, filtered, and the precipitate was washed with methanol (15 mL) and diethyl ether (30 mL) to yield 108 mg (22 %) of a bright red powder. Anal. calcd for C_20_H_18_CoF_2_N_4_O_2_⋅NO_3_⋅1.5H_2_O (%): C, 45.12; H, 3.98; N, 13.16. Found: C, 45.25; H, 3.76; N, 13.15. ^1^H NMR (500 MHz, [D_6_]DMSO) *δ*=8.89 (s, 2 H), 8.44–8.34 (m, 2 H), 7.50 (dd, *J*=6.4, 3.2 Hz, 2 H), 7.46 (d, *J*=7.7 Hz, 2 H), 7.28–7.18 (m, 2 H), 6.63–6.53 (m, 2 H), 3.04 (s, 6 H). ^19^F NMR (470 MHz, [D_6_]DMSO) *δ=−*133.26 (d, J_F‐H_=11 Hz, 2F). IR (KBr, cm^−1^): 3078 br w, 1617 s, 1543 m, 1448 m, 1370 m, 1228 m, 1191 s, 735 s. HRMS‐ESI (positive ion mode, CH_3_CN): *m*/*z* calcd for [C_20_H_18_CoF_2_N_4_O_2_]^+^: 443.0724; found: 443.0714.

**[Co(ATS)(DMAP)_2_]NO_3_ (3)**: ATS (200 mg, 0.85 mmol) was dissolved in methanol (8 mL) and Co(NO_3_)_2_⋅6 H_2_O (250 mg, 0.85 mmol) was added, resulting in a green suspension that slowly turned black. The mixture was heated at reflux for 1 h, after which DMAP (1.4 g, 11.4 mmol) was added. The reflux was continued for 16 h, and then the resulting dark red suspension was allowed to cool to room temperature, filtered, and the precipitate was washed with methanol (30 mL) and diethyl ether (30 mL) to yield 400 mg (78 %) of a dark red powder. Anal. calcd for C_20_H_30_CoN_10_S_2_⋅NO_3_⋅2.2H_2_O (%): C, 37.82; H, 5.46; N, 24.26. Found: C, 38.02; H, 5.49; N, 23.85. ^1^H NMR (400 MHz, [D_6_]DMSO) *δ*=7.66 (s, 4 H), 7.60 (d, *J*=6.9 Hz, 4 H), 6.54 (d, *J*=6.9 Hz, 4 H), 2.97 (s, 12 H), 2.59 (s, 6 H). IR (KBr, cm^−1^): 3417 w, 3286 m, 3178 m, 2913 w, 1626 s, 1539 m, 1439 s, 1378 m, 1230 s, 1060 w, 1017 m. HRMS‐ESI (positive ion mode, CH_3_CN): *m*/*z* calcd for [C_20_H_30_CoN_10_S_2_]^+^: 533.1423; found: 533.1433.

**Cyclic voltammetry**: Electrochemical measurements were carried out in the same manner as reported previously.^[25b]^ Experiments were conducted using a Pine WaveNow potentiostat with a three‐electrode setup consisting of a glassy carbon working electrode, a platinum counter electrode, and an Ag wire quasi‐reference electrode. Complexes were dissolved in anhydrous DMF with 0.10 M [Bu_4_N][PF_6_] (TBAP) as the supporting electrolyte. Potentials were referenced using an internal standard of the ferrocene/ferricenium couple at 0.45 V vs. the saturated calomel electrode (SCE).^[33]^ The sample cell was deoxygenated by bubbling nitrogen gas through the solution prior to analysis and maintained under a blanket of nitrogen during the experiment.

**Yeast strains**: *Candida albicans* (ATCC 90028, NCCLS 11), *Cryptococcus neoformans* (ATCC 208821, H99 type strain), *Candida tropicalis* (ATCC 750, type strain), *Candida glabrata* (ATCC 90030, NCCLS 84) and *Cryptococcus deuterogattii* (ATCC 32609, type strain) were obtained from the American Type Culture Collection (ATCC). *Cryptococcus deuterogattii* (CBS 7750), *Candida auris* (CBS 10913) and *Candida auris* (CBS 12373) were obtained from the CBS‐KNAW culture collection. The strains were maintained on glycerol/YPD (yeast extract‐peptone dextrose) broth (20:80 *v*/*v*) at −80 °C.

**Antifungal minimum inhibitory concentration (MIC) assay**: The cobalt complexes and antifungal control compounds were serially diluted in 50 μL of Yeast Nitrogen Broth (YNB; BD, Cat No. 233520 supplemented with 2 % glucose and 10 mM ammonium sulfate) two‐fold across the wells of non‐binding surface (NBS) 96‐well plates (Corning; Cat. No. 3641). Plates were set up in quadruplicate for each strain tested, and two biological replicates were conducted on separate assay days.

Yeast strains were cultured from glycerol onto YPD (Becton Dickinson 242720) agar at 30 °C for 72 h. For each strain, a minimum of five single colonies were taken from the agar plate and dissolved in sterile water and adjusted to form a yeast suspension of 1×10^6^ to 5×10^6^ CFU mL^−1^ (as determined by OD_530_). The suspension was subsequently diluted into YNB media, and added to each well of the compound containing plates, giving a final cell density of 2.5×10^3^ CFU mL^−1^ and a total volume of 100 μL. Plates were covered and incubated at 35 °C for 36 h without shaking.

Growth inhibition of all strains was determined visually where the MIC was recorded as the lowest compound concentration with no visible growth. Fluconazole (Sigma F8929), ketoconazole (Sigma K1003) and micafungin (Sigma SML2288) were used as internal positive inhibitor controls for every strain tested.

**Cytotoxicity assay**: HEK293 ATCC CRL‐1573 human embryonic kidney cells suspended in DMEM media (Gibco; 11330332) supplemented with 10 % FBS (GE; SH30084.03) and 100 U mL^−1^ each penicillin/streptomycin (Invitrogen; 15070063) were counted manually in a Neubauer haemocytometer and seeded into 384‐well, black wall, clear bottom tissue culture plates (Corning; Cat. No. 3712) at 5000 cells per well in a volume of 20 μL. Manually, 20 μL of each compound dilution was plated in duplicate on the cells, for a final concentration range of 0.8–100 μM. The cells were incubated together with the compounds for 20 h at 37 °C, 5 % CO_2_.

Cytotoxicity (or cell viability) was measured by fluorescence, ex: 560/10 nm, em: 590/10 nm (F560/590), after addition of 5 μL of 25 μg mL^−1^ resazurin (2.3 μg mL^−1^ final concentration; Sigma R7017) and after further incubation for 3 h at 37 °C in 5 % CO_2_, using media only as negative control and cells without inhibitors as positive control. CC_50_ (concentration at 50 % cytotoxicity) were calculated by curve fitting the inhibition values vs. log(concentration) of four replicates using a sigmoidal dose‐response function, with variable fitting values for bottom, top and slope using Prism 8. Tamoxifen (Sigma T5648) was used as internal control on each plate.

**Haemolysis assay**: Human whole blood (Australian Red Cross Blood Service) was washed three times with three volumes of 0.9 % NaCl and resuspended in a concentration of 0.5×10^8^ cells mL^−1^, determined by manual cell count in a Neubauer haemocytometer. Washed cells were added to compound containing plates (384‐well round bottom polypropylene plates, Corning 3657) for a final volume of 50 μL, shaken for 10 min and incubated for 1 h at 37 °C, without shaking. After incubation, the plates were centrifuged at 1000 g for 10 min to pellet cells and debris, 25 μL of the supernatant was then transferred to reading plates (384‐well flat bottom polystyrene plates, Corning CLS3680), with haemolysis determined by measuring the supernatant absorbance at 405 mm (OD_405_), using cells without inhibitors as negative control and cells with 1 % Triton X‐100 (Sigma T8787) as positive control. HC_10_ and HC_50_ (concentration at which 10 and 50 % haemolysis is induced, respectively) were calculated by curve fitting the inhibition values vs. log(concentration) of four replicates using a sigmoidal dose–response function with variable fitting values for top, bottom and slope using Prism 8. Melittin (Sigma M2272) was used as positive haemolytic control on each plate. Human ethics approval from the University of Queensland Institutional Human Research Ethics Committee was obtained for use of human blood for haemolysis studies (approval number 201400003).

**In vivo tox (moth)**: *G. mellonella* larvae were reared in controlled environmental room at Macquarie University, Sydney, Australia at 26 °C and 65 % humidity with a 12‐hours light/dark cycle. Larvae (≈200 mg) were individually injected with 10 μL of chemical into the last right proleg using a 100 μL syringe (Hamilton Ltd). The injection was done for compounds **1**–**3**. Each compound was dissolved in DMSO and diluted to final concentrations of 1 μM, 100 μM and 10 mM. We injected five larvae for each dilution of each compound. Larvae injected with different dilutions of DMSO (10^−1^, 10^−3^ and 10^−5^) were included as negative controls. Following injection, the larvae were incubated at 26 °C and monitored every 24 h for 4 days. Larval performance was assessed according to the *G. mellonella* Health Index Scoring System.^[30]^ The experiments were repeated three separate times.

## Conflict of interest

The authors declare no conflict of interest.

## Supporting information

As a service to our authors and readers, this journal provides supporting information supplied by the authors. Such materials are peer reviewed and may be re‐organized for online delivery, but are not copy‐edited or typeset. Technical support issues arising from supporting information (other than missing files) should be addressed to the authors.

Supplementary
