# Supplementary material for: Nontoxic Cobalt(III) Schiff Base Complexes with Broad‐Spectrum Antifungal Activity
Source: Chemistry. 2020 Nov 24;27(6):2021–9. doi: 10.1002/chem.202003545 (PMC7855930; doi:10.1002/chem.202003545)
Supplement: Supplementary file 1 — Supplementary [file CHEM-27-2021-s001.pdf]

Chemistry–A European Journal

Supporting Information

Nontoxic Cobalt(III) Schiff Base Complexes with Broad-Spectrum Antifungal Activity

Angelo Frei,*[a] A. Paden King,[b] Gabrielle J. Lowe,[a] Amy K. Cain,[c] Francesca L. Short,[c] Hue Dinh,[c, d] Alysha G. Elliott,[a] Johannes Zuegg,[a] Justin J. Wilson,[b] and Mark A. T. Blaskovich*[a]

Table S1. Antifungal activity displayed as minimum inhibitory concentrations (MIC, µg/mL) of all compounds in this study against a panel of fungal strains.

	Candida albicans ATCC 90028	***Candida auris*** CBS 10913	***Candida auris*** CBS 12373	***Candida glabrata*** ATCC 90030	***Candida tropicalis*** ATCC 750	***Cryptococcus deuterogattii*** CBS 7750	***Cryptococcus deuterogattii*** ATCC 32609	***Cryptococcus neoformans*** ATCC 208821
**1**	0.38 - 0.76	0.19 – 0.38	0.19 – 0.38	48.7	0.19	0.76	0.38 – 0.75	0.19 - 0.38
**2**	0.72 - 2.87	0.72 - 1.43	1.43	5.73 – 11.5	0.36 – 0.72	2.87	1.43	1.43 - 2.87
**3**	0.93	0.23 - 0.46	0.46	29.7	0.23 - 0.46	0.93	0.93 – 1.86	0.46 – 0.93
**4**	>67.4	4.21	67.4	>67.4	8.42 – 16.8	33.7	16.8	16.8 - 33.7
**5**	64.6	8.07	8.07	64.6	16.1 – 32.3	8.07 – 16.1	8.07 – 16.1	32.3
**6**	69.4	0.03 – 0.54	17.3	>69.4	>69.4	>69.4	69.4	69.4
**7**	23.9 – 47.8	0.37 – 0.75	23.9	47.8	47.8	47.8	47.8	47.8
**L1**	>23.2	>23.2	>23.2	>23.2	>23.2	>23.2	>23.2	>23.2
**L2**	>33.2	>33.2	>33.2	>33.2	>33.2	>33.2	>33.2	>33.2
**L3**	>30.4	>30.4	>30.4	>30.4	>30.4	>30.4	>30.4	>30.4
**$Co(NO_3)_2$**	>29.1	>29.1	29.1	>29.1	>29.1	>29.1	>29.1	>29.1
**FCZ[a]**	2	16	>64	>64	>64	8	8 - 16	16
**KCZ[b]**	≤0.5	≤0.5	2	32	16	≤0.5 – 1	1	1-2
**MFG[c]**	≤0.5	<0.0.5	≤0.5	≤0.5	≤0.5	>64	>64	>64

[a]FCZ- Fluconazole; [b]KCZ – Ketoconazole; [c]MFG – Micafungin.

Table S2. MIC values of all compounds against the CO-ADD bacterial panel (µg/mL).

	**Sa[a]**	**Ec[b]**	**Kp[c]**	**Pa[d]**	**Ab[e]**
**1**	>32	>32	32	>32	>32
**2**	>32	>32	>32	>32	>32
**4**	4	>32	>32	>32	>32
**5**	16	>32	>32	>32	>32
**L1**	>32	>32	>32	>32	>32
**L2**	>32	>32	>32	>32	>32
**L3**	>32	>32	>32	>32	>32
**3**	>32	>32	>32	>32	>32
**6**	8	>32	>32	>32	>32
**7**	>32	>32	>32	>32	>32
**$Co(NO_3)_2$**	>32	>32	>32	>32	>32
**Col[i]**	-	0.125	0.25	0.25	0.25
**Van[j]**	1	-	-	-	-

[a]Sa -*Staphylococcus aureus* ATCC 43300 (MRSA); [b]Ec - *Escherichia coli* ATCC 25922; [c]Kp - *Klebsiella pneumoniae* ATCC 700603 (K6; ESBL SHV-18); [d]Pa - *Pseudomonas aeruginosa* ATCC 27853; [e]Ab - *Acinetobacter baumannii* ATCC 19606; [i]Colistin; [j] Vancomycin,

CO-ADD High Throughput Screening

Bacterial broth microdilution minimum inhibitory concentration (MIC) assay

Bacterial strains were cultured in Cation-adjusted Mueller Hinton broth (CAMHB; Bacto Laboratories 212322) at 37 °C overnight. A sample of each culture was then diluted 40-fold in fresh CAMHB and incubated at 37 °C for 1.5-3 h. The resultant mid-log phase cultures were diluted with CAMHB (CFU/mL measured by OD_{600}), then added to each well of the compound-containing plates (384-well non-binding surface (NBS) plates; Corning CLS3640), giving a cell density of 5×10^5 CFU/mL and a total volume of 50 µL. Plates were covered and incubated at 37 °C for 18 h without shaking. Inhibition of bacterial growth was determined measuring absorbance at 600 nm (OD_{600}), using media only as negative control and bacteria without inhibitors as positive control. MIC values were determined as the lowest concentration at which the growth was inhibited at ≥ 80%. Colistin sulfate (Sigma C4461) and vancomycin HCl (Sigma 861987) were used as positive inhibitor controls on each plate for Gram-negative and Gram-positive bacteria, respectively.

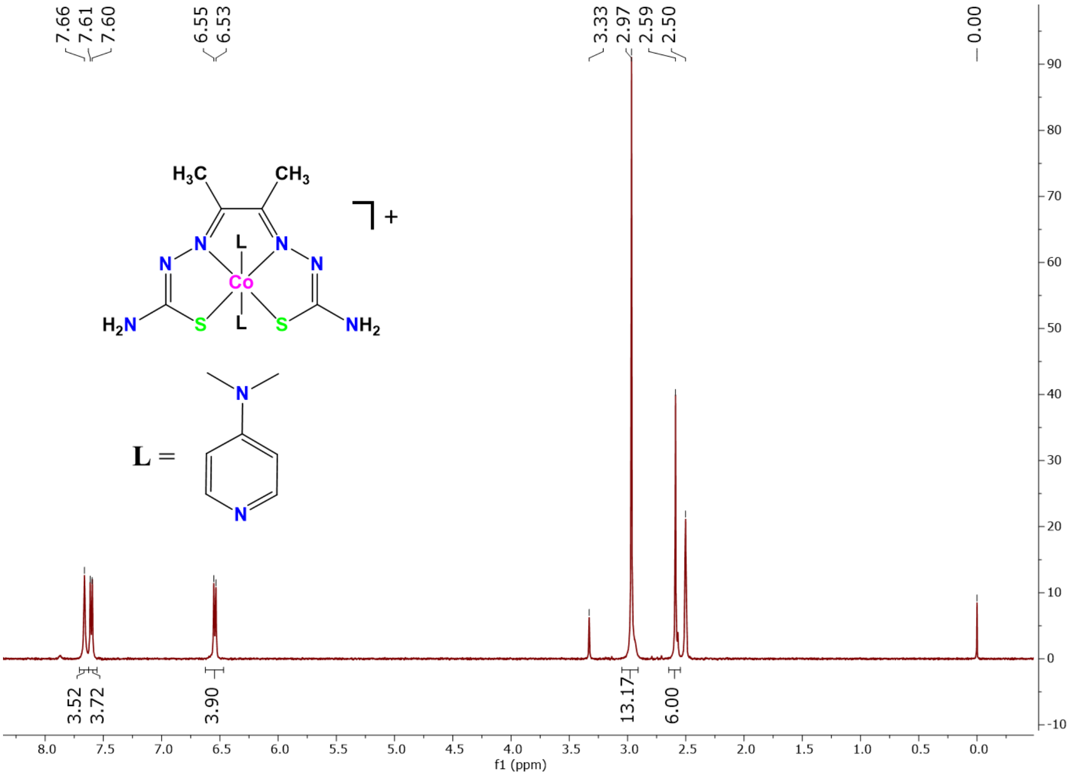

Figure S1. ^{1}H NMR (400 MHz) of $[Co(ATS)(DMAP)_2]NO_3$ (**3**) in DMSO-d6.

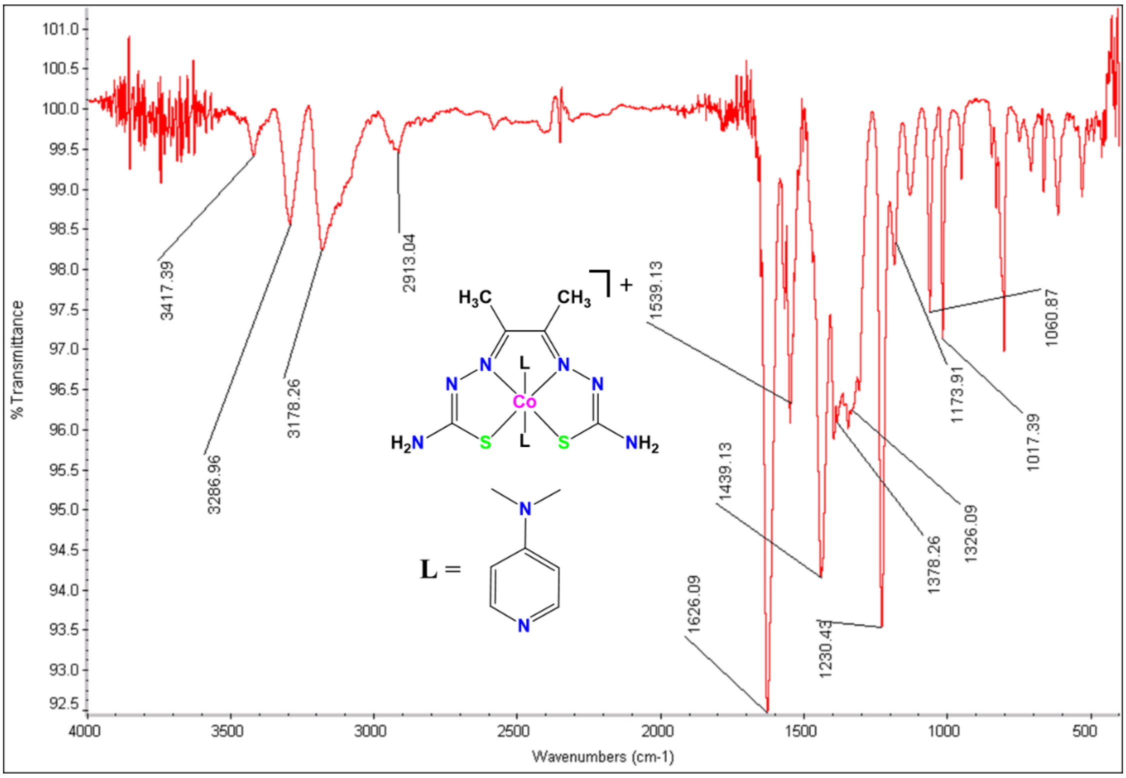

Figure S2. IR (KBr, cm-1) of [Co(ATS)(DMAP)2]NO3 (**3**).

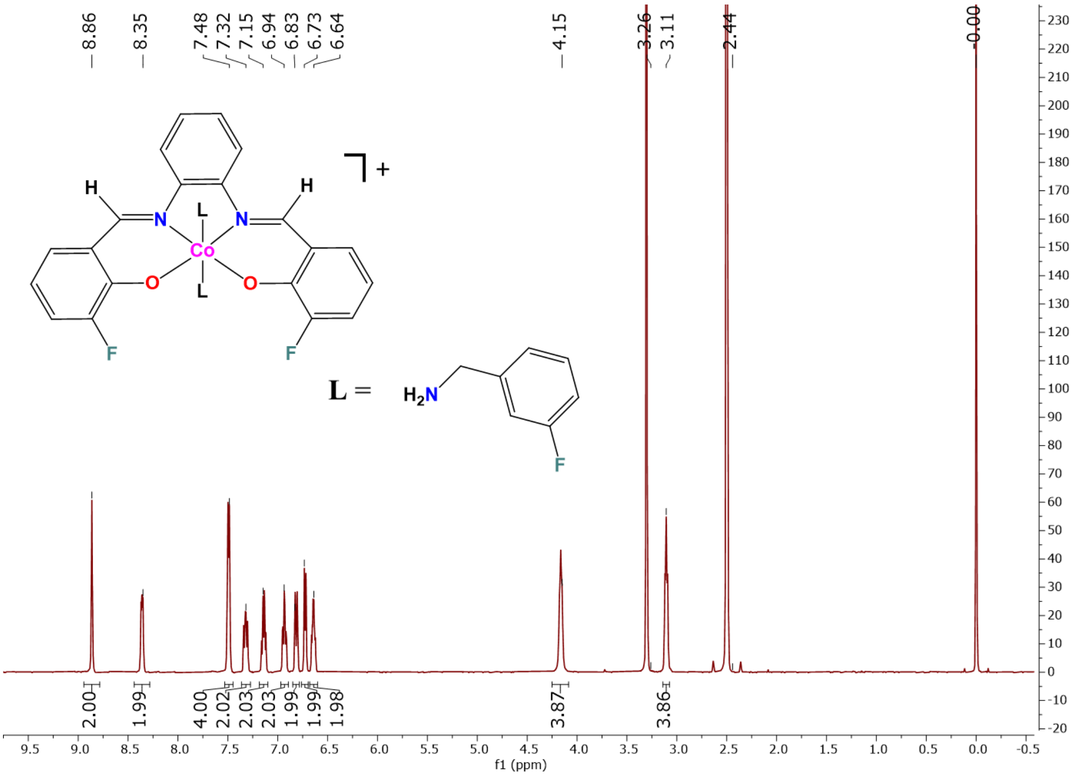

Figure S3. ^{1}H NMR (500 MHz) of [Co(3F-salophen)(3F-BnNH2)2]NO3 (**6**) in DMSO-d6.

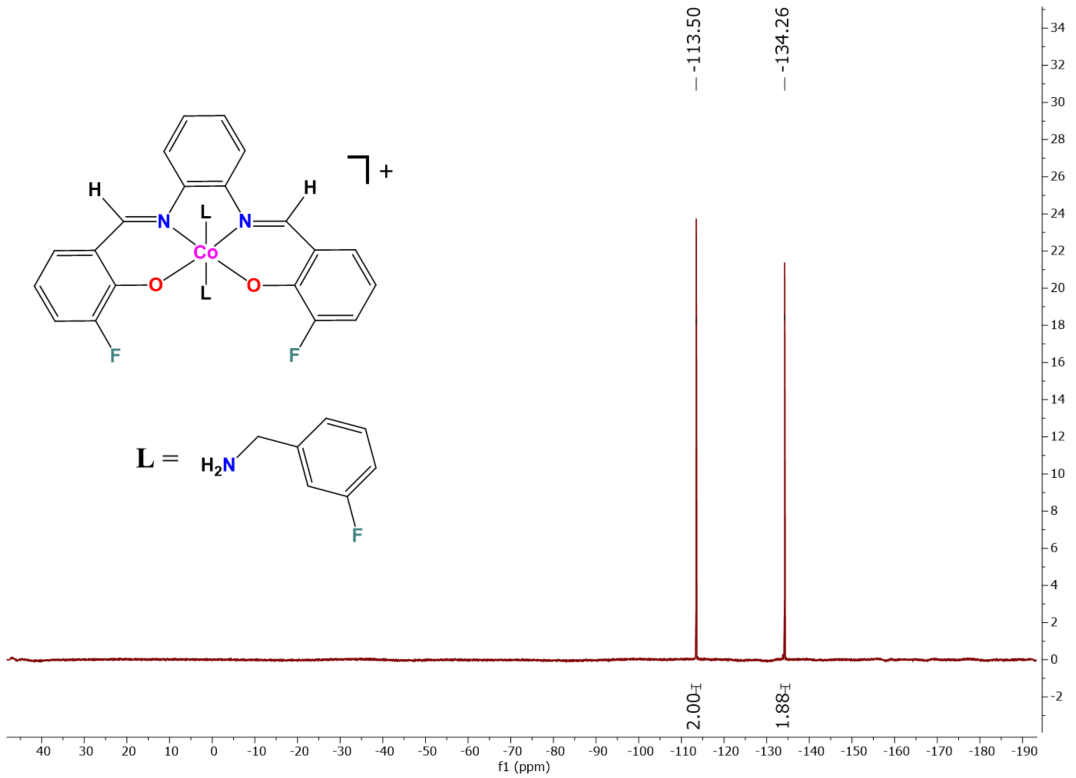

Figure S4. ^{19}F NMR (470 MHz) of [Co(3F-salophen)(3F-BnNH$_2$)$_2$]NO$_3$ (**6**) in DMSO-d6.

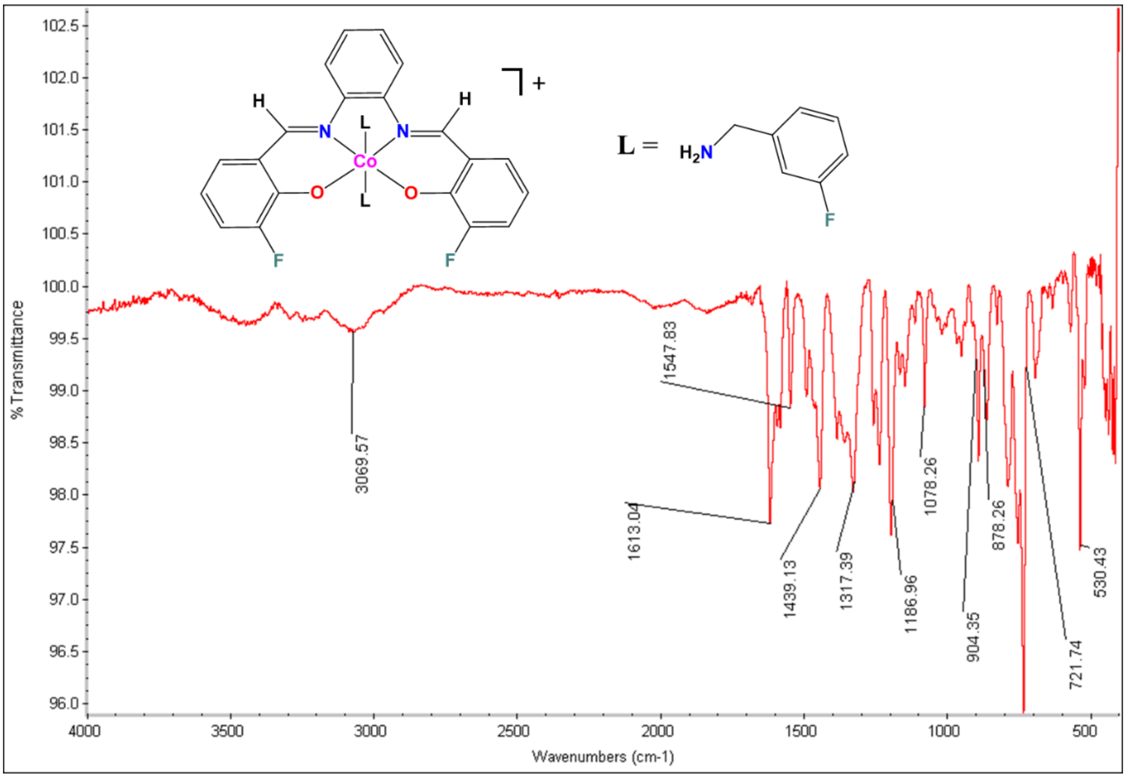

Figure S5. IR (KBr, cm^{-1}) of [Co(3F-salophen)(3F-BnNH$_2$)$_2$]NO$_3$ (**6**).

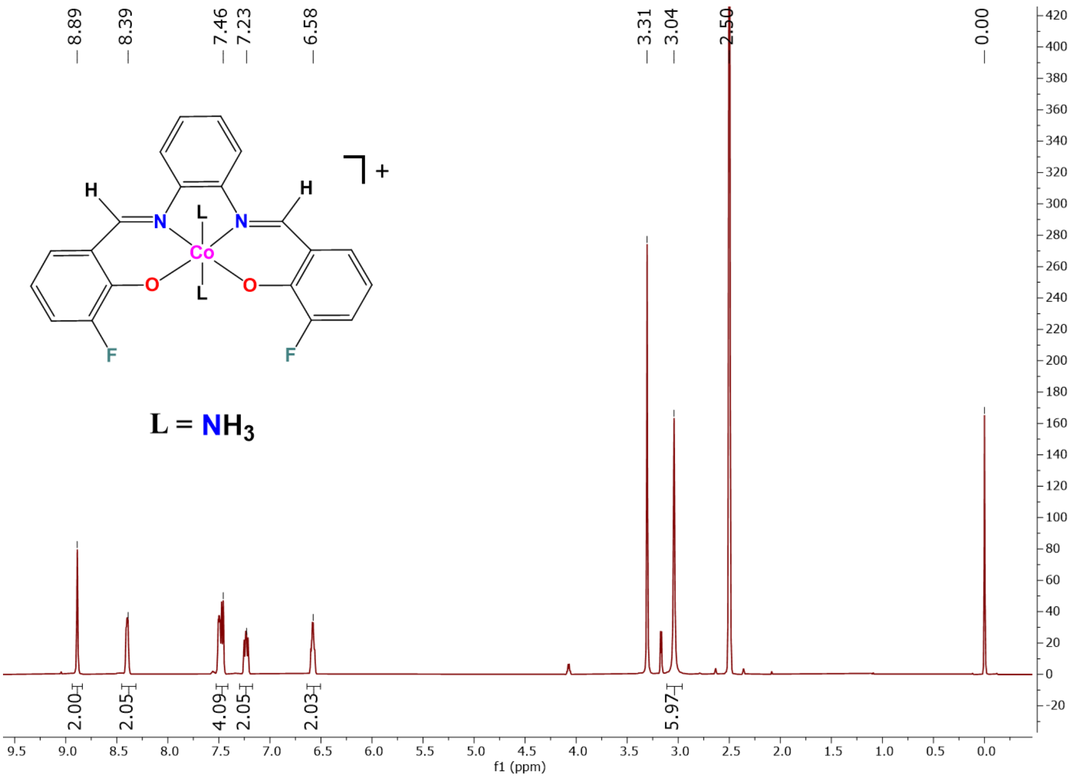

Figure S6. ^{1}H NMR (500 MHz) of $[Co(3F\text{-}salophen)(NH_3)_2]NO_3$ (**7**) in DMSO-d6.

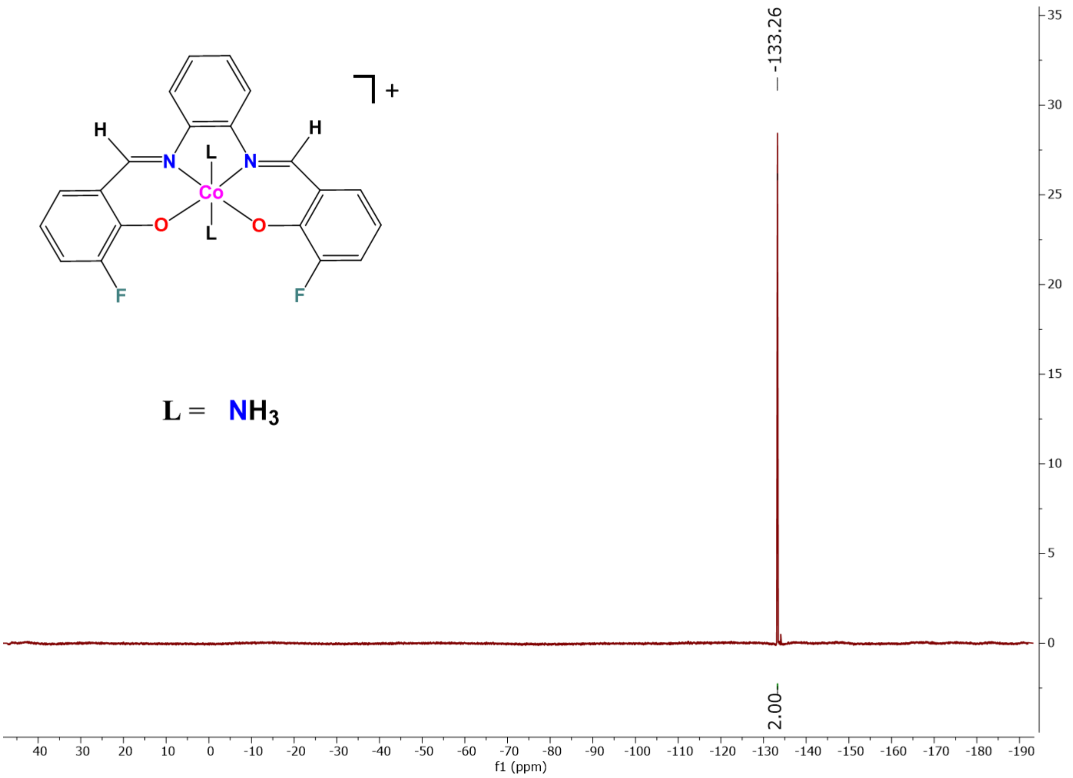

Figure S7. ^{19}F NMR (470 MHz) of $[Co(3F\text{-}salophen)(NH_3)_2]NO_3$ (**7**) in DMSO-d6.

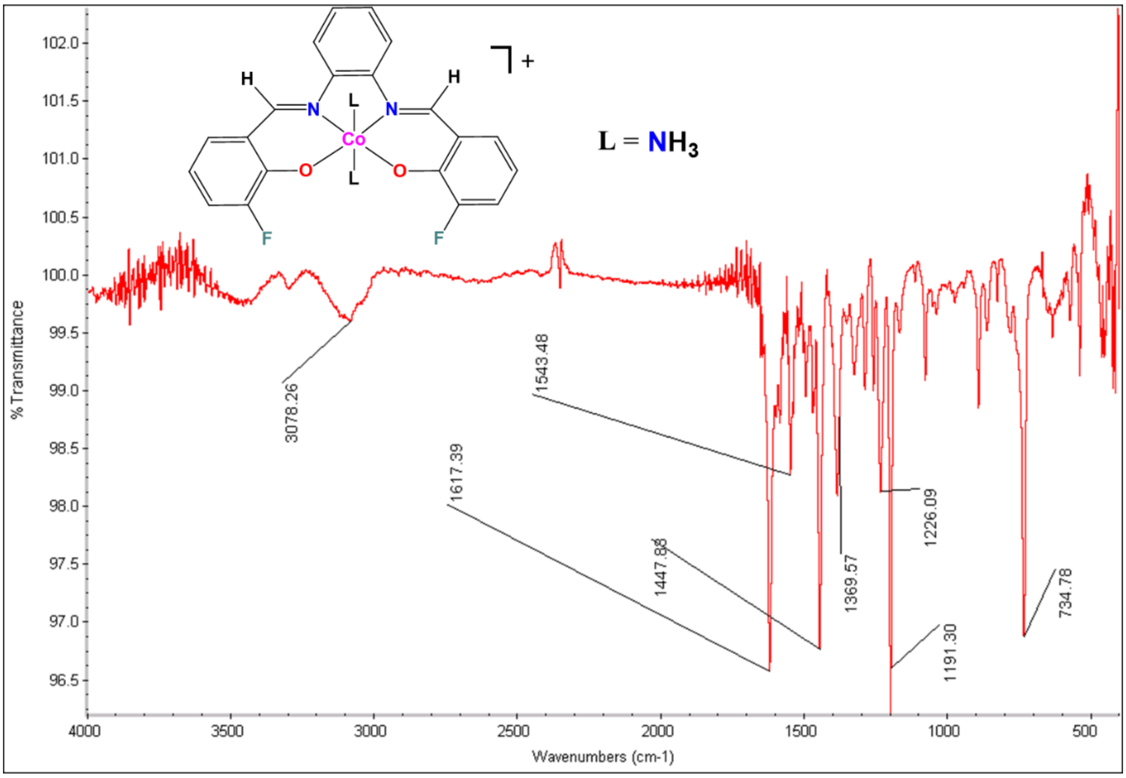

Figure S8. IR (KBr, cm^{-1}) of $[Co(3F\text{-}salophen)(NH_3)_2]NO_3$ (**7**).

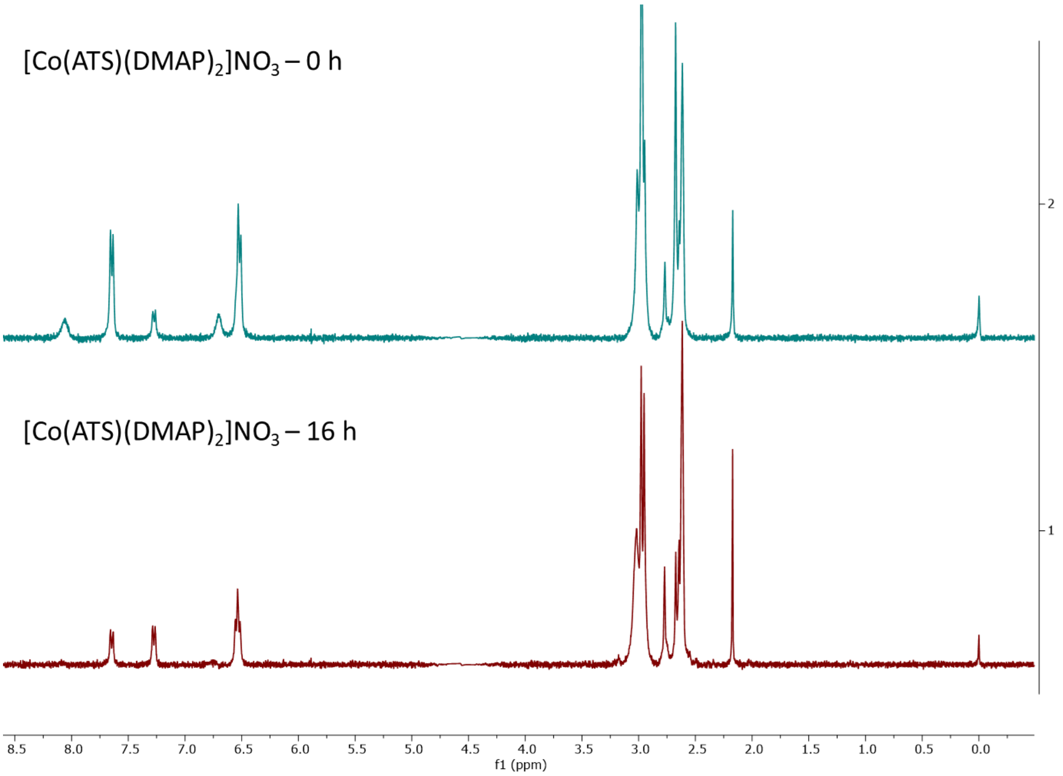

Figure S9. ^1H NMR (400 MHz) of $[Co(ATS)(DMAP)_2]NO_3$ (**3**) in a mixture of DMSO-d6 and H_2O (50:50). The H_2O peak at 4.58 ppm was suppressed using the suppression function in MNOVA.

Acquisition Parameter

Source Type	ESI	Ion Polarity	Positive	Set Nebulizer	0.8 Bar
Focus	Not active			Set Dry Heater	180 °C
Scan Begin	100 m/z	Set Capillary	4500 V	Set Dry Gas	5.0 l/min
Scan End	1000 m/z	Set End Plate Offset	-500 V	Set Divert Valve	Source

Generate Molecular Formula Parameter

Formula, min.		
Formula, max.		
Measured m/z	Tolerance	Charge
Check Valence	Minimum	Maximum
Nirogen Rule	Electron Configuration	
Filter H/C Ratio	Minimum	Maximum
Estimate Carbon		

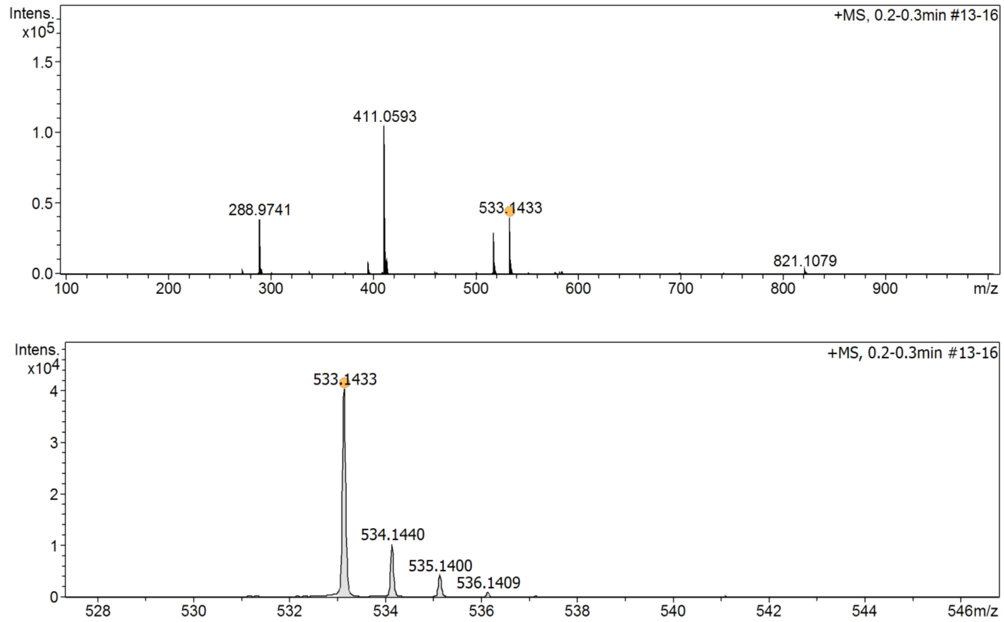

Meas. m/z	#	Ion Formula	m/z	err [ppm]	mSigma	# Sigma	Score	rdb	e⁻ Conf	N-Rule
533.1433	1	C20H30CoN10S2	533.1423	-1.9	11.0	1	100.00	11.0	odd	-

Figure S10. HR-ESI MS of compound **3** (CH_3CN).

Acquisition Parameter

Source Type	ESI	Ion Polarity	Positive	Set Nebulizer	0.8 Bar
Focus	Not active			Set Dry Heater	180 °C
Scan Begin	100 m/z	Set Capillary	4500 V	Set Dry Gas	5.0 l/min
Scan End	1000 m/z	Set End Plate Offset	-500 V	Set Divert Valve	Source

Generate Molecular Formula Parameter

Formula, min.		
Formula, max.		
Measured m/z	Tolerance	Charge
Check Valence	Minimum	Maximum
Nirogen Rule	Electron Configuration	
Filter H/C Ratio	Minimum	Maximum
Estimate Carbon		

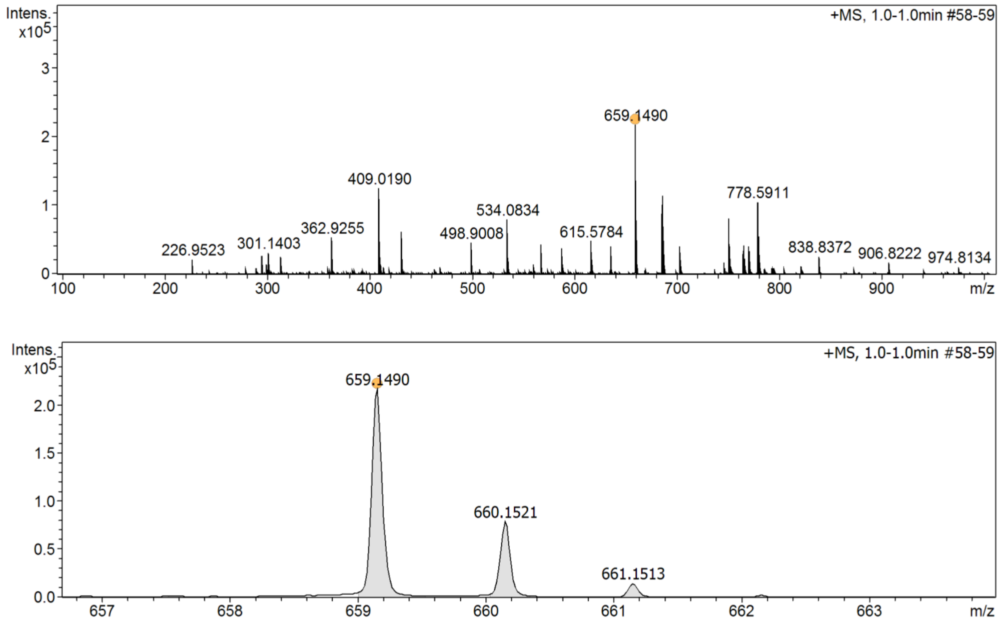

Meas. m/z	#	Ion Formula	m/z	err [ppm]	mSigma	# Sigma	Score	rdb	e⁻ Conf	N-Rule
659.1490	1	C34H28CoF4N4O2	659.1475	-2.3	13.6	2	100.00	21.0	odd	-

Figure S11. HR-ESI MS of compound **6** (CH_3CN + COOH + NaOH).

Acquisition Parameter

Source Type	ESI	Ion Polarity	Positive	Set Nebulizer	0.8 Bar
Focus	Not active			Set Dry Heater	180 °C
Scan Begin	100 m/z	Set Capillary	4500 V	Set Dry Gas	5.0 l/min
Scan End	1000 m/z	Set End Plate Offset	-500 V	Set Divert Valve	Source

Generate Molecular Formula Parameter

Formula, min.		
Formula, max.		
Measured m/z	Tolerance	Charge
Check Valence	Minimum	Maximum
Nirogen Rule	Electron Configuration	
Filter H/C Ratio	Minimum	Maximum
Estimate Carbon		

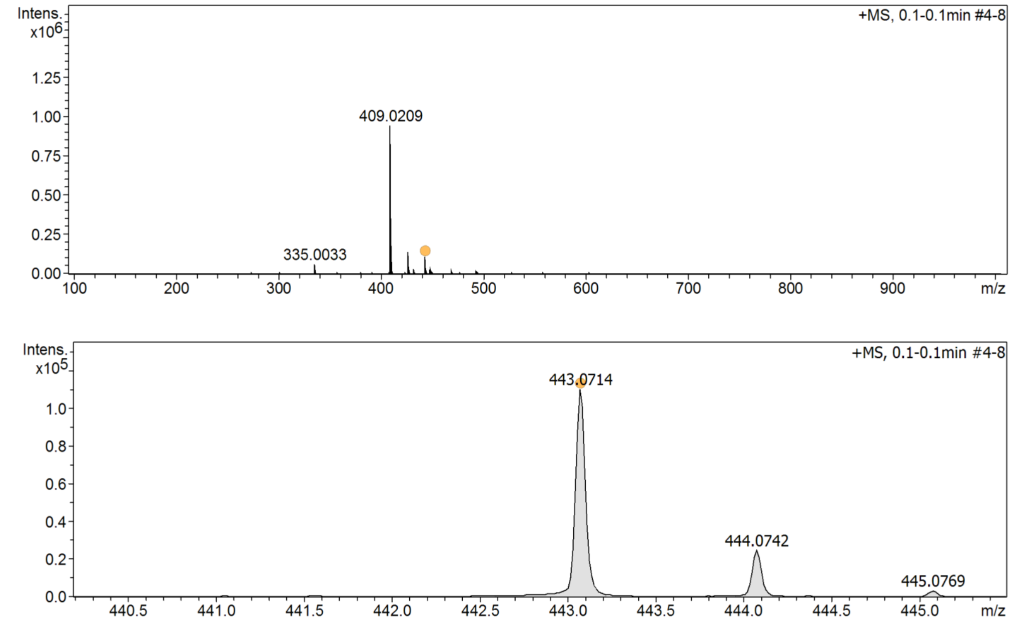

Meas. m/z	#	Ion Formula	m/z	err [ppm]	mSigma	# Sigma	Score	rdb	e⁻ Conf	N-Rule
443.0714	1	C20H18CoF2N4O2	443.0724	2.4	5.6	2	56.39	13.0	odd	-

Figure S12. HR-ESI MS of compound **7** (CH_3CN).

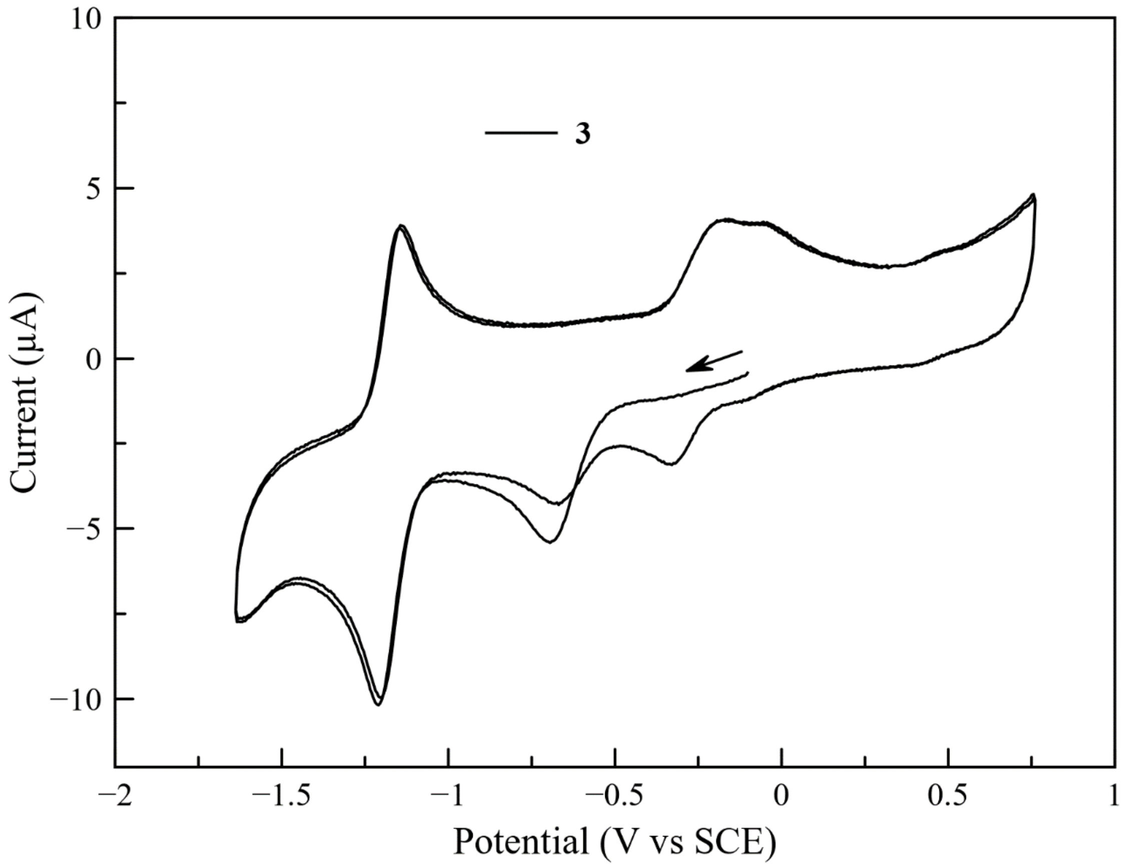

Figure S13. Cyclic voltammogram of **3.** This experiment was performed at 25 °C in DMF solution with 0.10 M TBAP electrolyte at a scan rate of 100 mV/s. The potential is referenced to SCE on the basis of the position of the Fc/Fc^{+} couple as an internal standard.

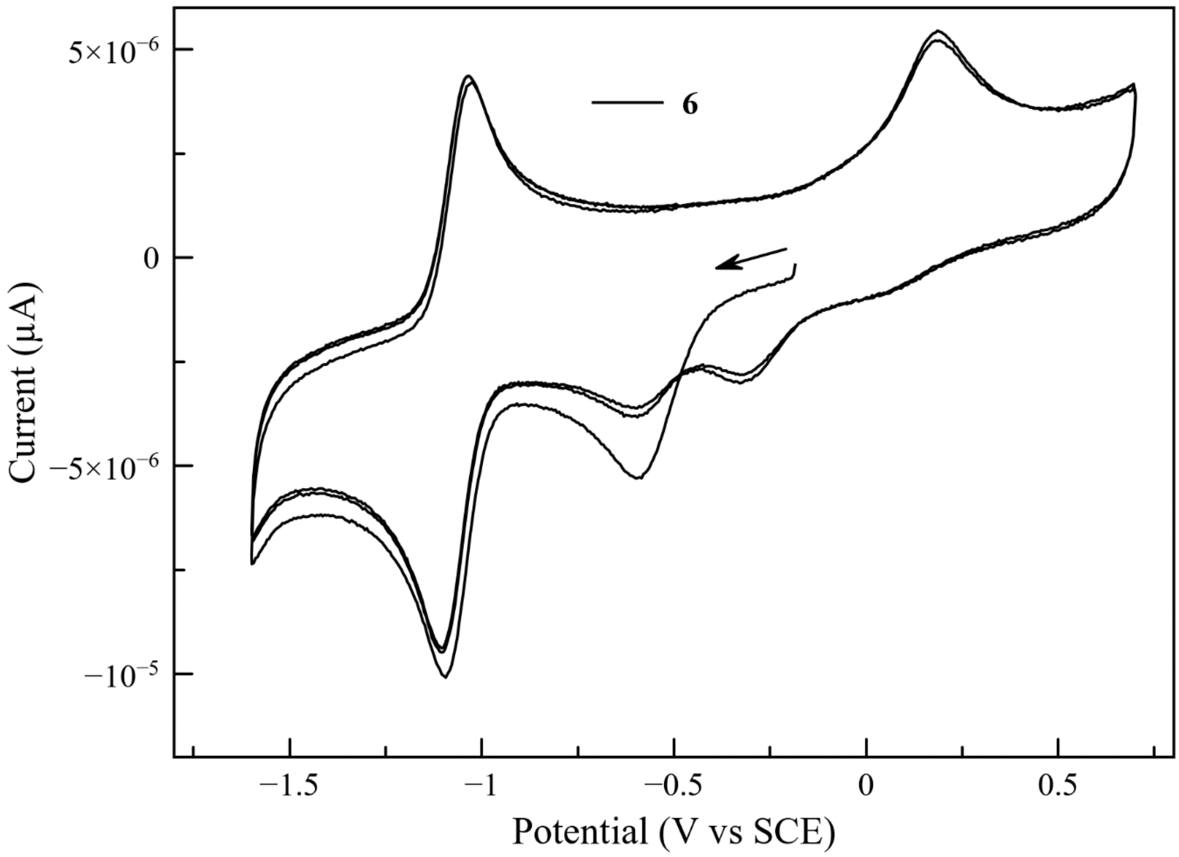

Figure S14. Cyclic voltammogram of **6.** This experiment was performed at 25 °C in DMF solution with 0.10 M TBAP electrolyte at a scan rate of 100 mV/s. The potential is referenced to SCE on the basis of the position of the Fc/Fc^{+} couple as an internal standard.

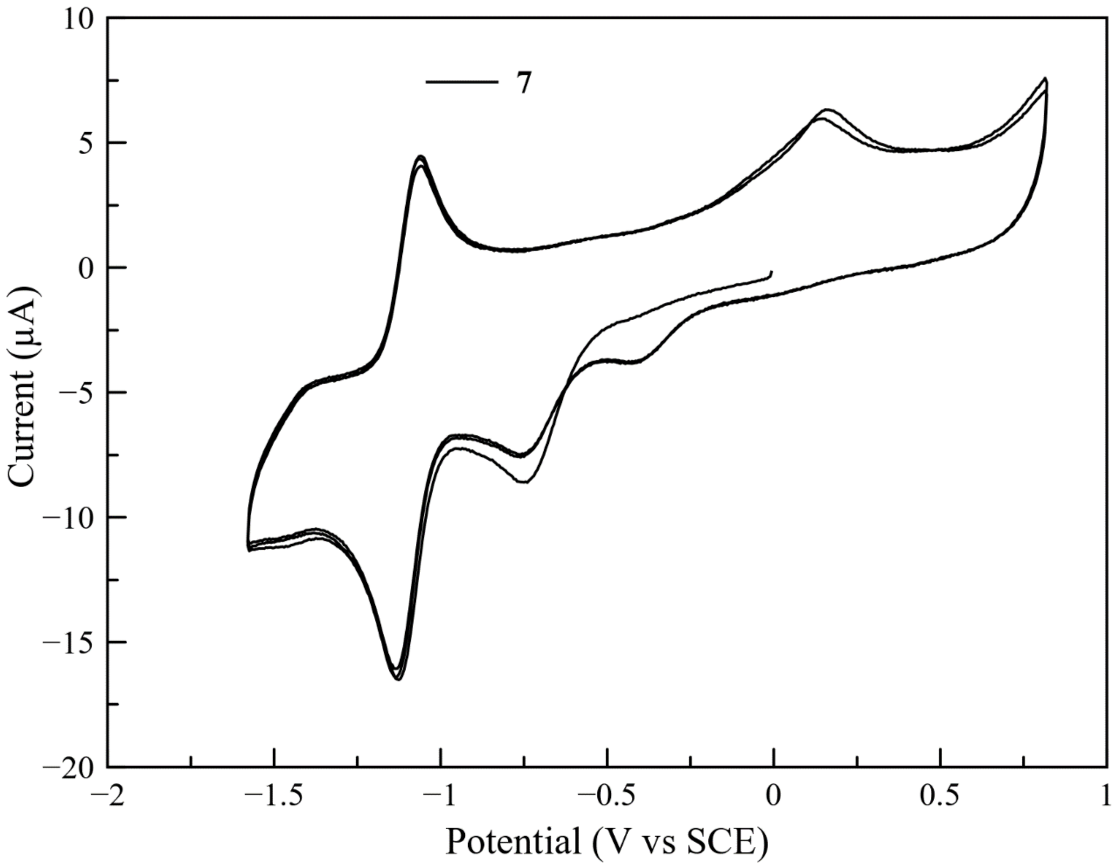

Figure S15. Cyclic voltammogram of **7.** This experiment was performed at 25 °C in DMF solution with 0.10 M TBAP electrolyte at a scan rate of 100 mV/s. The potential is referenced to SCE on the basis of the position of the Fc/Fc^+ couple as an internal standard.
